# Exploring *Rhamnus alaternus* Polysaccharides: Extraction, Characterization, and Analysis of Antioxidant and Antimicrobial Properties

**DOI:** 10.3390/polym16223180

**Published:** 2024-11-15

**Authors:** Souha Chokri, Sonia Ben Younes, Ali Ellafi, Sami Mnif, Eduardo Alberto López-Maldonado, Ahmed Slaheddine Masmoudi

**Affiliations:** 1 Laboratory of Biotechnology and Biogeoresources Valorization, Higher Institute of Biotechnology of Sidi Thabet (ISBST), LR11ES31, Ariana 2020, Tunisia; souhachokri01@gmail.com (S.C.); ahmedsleheddine.masmoudi@isbst.uma.tn (A.S.M.); 2 Faculty of Sciences of Gafsa, University of Gafsa, Campus Sidi Ahmed Zarroug, Gafsa 2112, Tunisia; ali_lafi160@yahoo.fr; 3 Laboratory of Population Health, Environmental Aggressors and Alternative Therapies (LR24ES10), Faculty of Medicine of Tunis, University of Tunis El Manar, Tunis 1068, Tunisia; 4 Laboratory of Analysis, Treatment and Valorization of Environment Pollutants and Product, Faculty of Pharmacy, Monastir University, Monastir 5000, Tunisia; 5 Laboratory of Molecular and Cellular Screening Processes, Centre of Biotechnology of Sfax, Sfax 3018, Tunisia; sami.mnif@gmail.com; 6 Faculty of Chemical Sciences and Engineering, Autonomous University of Baja California, Tijuana 22424, Mexico

**Keywords:** *Rhamnus alaternus*, stems and leaves, water-soluble polysaccharides, antioxidant activities, antimicrobial activities

## Abstract

In the present study, polysaccharides were isolated from the leaves (WSPRaL) and stems (WSPRaS) of *Rhamnus alaternus* with yields of 3% and 3.25% for WSPRaS and WSPRaL, respectively. Crude WSPRaL was composed of proteins (260.740 ± 0.98 µg/mg), lipids (53.34 ± 2.38 µg/mg), total sugars (482.716 ± 3.02 µg/mg), and reducing sugars (420.240 ± 1.68 µg/mg). In contrast, WSPRaS contained proteins (269.629 ± 1.48 µg/mg), lipids (13.33 ± 0.28 µg/mg), total sugars (569.135 ± 3.82 µg/mg), and reducing sugars (531.732 ± 2.59 µg/mg). FTIR, TLC, and HPLC analyses revealed that the carbohydrate fraction of WSPRaS consisted mainly of glucuronic acid, glucose, galactose, xylose, mannose, and arabinose, whereas WSPRaL consisted of galacturonic acid, sucrose, glucose, rhamnose, xylose, mannose, and arabinose. Scanning electron microscopy (SEM) analysis was used to determine the microstructure of the water-soluble polysaccharides. The physicochemical properties were evaluated using Fourier transform infrared (FT-IR) spectroscopy and ultraviolet‒visible (UV‒visible) absorption spectroscopy. The total antioxidant activities of the crude polysaccharides were evaluated using various assays: DPPH radical scavenging (IC_50_ WSPRaL = 615 ± 2.05 µg/mL, IC_50_ WSPRaS = 628 ± 2.38 µg/mL), ABTS radical scavenging (470 ± 5.78 µg/mL and 559 ± 4.32 µg/mL for WSPRaL and WSPRaS, respectively), reducing power (IC_50_ WSPRaS = 141.76 ± 3.16 µg/mL, IC_50_ WSPRaL = 203.89 ± 1.07 µg/mL), and chelating capacity (IC_50_ WSPRaS = 225 ± 1.75 µg/mL, IC_50_ WSPRaL = 219 ± 2.51 µg/mL). In addition, the antibacterial and biofilm inhibitory activities of both polysaccharides were tested against pathogenic strains, and the polysaccharides significantly inhibited plant growth. Overall, the results indicate that the crude polysaccharides extracted from *R. alaternus* are promising for use as functional and bioactive ingredients in the food and nutraceutical industries. These results highlight the potential of both polysaccharides as natural products in various sectors, including food, cosmetics, pharmaceuticals, and medicine, due to their significant biological properties.

## 1. Introduction

In recent years, the global healthcare landscape has been confronted with an alarming increase in microbial infections, particularly in immunocompromised patients and those undergoing surgery. This increase is due primarily to the indiscriminate use of antimicrobial agents in medical and veterinary practices, leading to the emergence of resistant microbial strains [1]. Consequently, there is an urgent need to redirect scientific research toward the exploration of novel biological molecules to fight against this pressing problem. Overreliance on antibiotics has significantly contributed to the development of bacterial resistance, setting a significant challenge for the effective treatment of bacterial infections. This resistance highlights the critical need for alternative therapeutic options, leading to a shift in focus toward natural products, particularly medicinal plants [2]. Medicinal plants have served for a long time as the cornerstone of traditional medical systems, providing a rich repository of bioactive compounds with deep therapeutic properties [3]. Among these botanical wonders, aromatic and medicinal plants, which span pharmaceuticals, medicine, cosmetology, and agriculture, have attracted considerable interest for their diverse biological activities [4]. Indeed, the wealth of natural products found in medicinal plants has demonstrated remarkable efficacy in treating various infectious diseases [5]. This resurgence of natural remedies can be attributed to growing concerns about the adverse effects associated with synthetic drugs, coupled with environmental sustainability considerations [3].

Recent research has focused on plant polysaccharides, recognizing them as central biomacromolecules with diverse applications in the food and biomedical industries [6]. Polysaccharides are ubiquitous in nature and offer a number of biological and physiological benefits, including biodegradability, nontoxicity, and antimicrobial properties [7,8]. The widespread use of polysaccharides in the food and pharmaceutical sectors highlights their importance in various biological processes, ranging from cell‒cell communication to immune modulation [9]. Furthermore, their therapeutic potential extends to various medical fields, including ulcer protection, antitumor and antiviral activities [10,11,12]. Among the myriad medicinal plants, *R. alaternus* L. (Rhamnaceae) has attracted attention for its extensive use in traditional medicine in Tunisia and North Africa [13]. Despite its historical importance, scientific research on the chemical and biological properties of *R. alaternus* remains limited.

Given this knowledge gap, the present study aimed to elucidate the physicochemical and biological properties of polysaccharides extracted from the leaves and stems of *R. alaternus*. Furthermore, this research endeavored to perform spectral characterization of the extracted water-soluble polysaccharides and to evaluate their antioxidant, antimicrobial, and antibiofilm activities in vitro. By exploring the therapeutic potential of *R. alaternus*-derived polysaccharides, this study aims to contribute to the growing body of evidence supporting the integration of natural remedies into modern medical practice, thereby addressing the emerging challenges of microbial resistance and environmental sustainability.

## 2. Materials and Methods

### 2.1. Chemicals

2,2-Diphenyl-1-picrylhydrazyl (DPPH), 2,2′-azino-bis(3-ethylbenzothiazoline-6-sulfonic acid (ABTS), butylated hydroxytoluene (BHT), potassium ferricyanide, ferric chloride, and crystal violet (c3886) were purchased from Sigma‒Aldrich (Deisenhofen, Germany). Monosaccharide standards (D-glucose (Glc), D-galactose (Gal), D-galacturonic acid (GalA), L-rhamnose (Rha), D-mannose (Man), L-arabinose (Ara), sucrose (Suc) (all these sugars were purchased from Sigma Chemical GmbH, Deisenhofen, Germany), xylose (Xyl) (United States Pharmacopeia (USP), Reference Standard), and glucuronic acid (GlcA) (Fluka Chemika GmbH, Steinheim, Germany) were used for reference in TLC and HPLC.

### 2.2. Raw Materials

The plant material used in this study was the leaves and stems of *R. alaternus* (Rhamnaceae). *R. alaternus* is widespread throughout Tunisia. Leaves and stems were collected in March 2022 in the arid region of Fernana (36°40′13.0″ N 8°43′18.4″ E), air-dried in the shade at room temperature, and ground into a fine powder. The dried powder was then treated with ethanol for 6 h to remove interfering components such as lipids, pigments, and polyphenols. The delipidated powder was then air dried and stored at −20 °C until use [6].

### 2.3. Bacterial Strains

This study focused on six reference bacterial strains: *Bacillus cereus* (ATCC 14579), *Escherichia coli* (ATCC 35218), *Enterobacter* sp. (ATCC 21754), *Klebsiella pneumoniae* (ATCC 13883), *Staphylococcus aureus* (ATCC 25923), and *Pseudomonas aeruginosa* (ATCC 27853). These strains were subsequently grown in Mueller–Hinton liquid media.

### 2.4. Extraction of Water-Soluble Polysaccharides

Crude polysaccharides were extracted from the leaves and stems of *R. alaternus* according to the method of Hamed et al. (2020) [6], with slight modifications. The delipidated powder of *R. alaternus* (stems and leaves) was extracted twice with seven volumes of distilled water at 90 °C for 4 h, then filtered. The concentrated liquid was precipitated with 95% (*v*/*v*) ethanol at 4 °C for 24 h to isolate the polysaccharides. The mixture was then centrifuged (4500× *g*) for 15 min using a refrigerated centrifuge (Jouan KR25i, Thermo Scientific, Waltham, MA, USA). The water phase, which was obtained by removing the proteins using trichloroacetic acid (TCA) (5%), was subjected to dialysis (cutoff = 1 kDa) against distilled water for three days to remove salts and impurities. The dialysate was then concentrated by rotary evaporation (rotary evaporator, Heidolph, Schwabach, Germany) and subsequently lyophilized. The extraction yield (Equation (1)) of the crude water-soluble polysaccharides was calculated according to Li and Zhang (2009) [14] using the following formula:

Extraction yield (%) = (P_1_/P_0_) × 100 (1)

where **P_1_** is the weight of polysaccharide extract (g) after evaporation, and **P_0_** is the initial weight of dry powder of the sample (g).

### 2.5. Spectral Characterization of Water-Soluble Polysaccharides

The water-soluble polysaccharides (WSPs) extracted from the stems (WSPRaS) and leaves (WSPRaL) of *R. alaternus* were subjected to extensive spectral, microscopic, and chromatographic analyses.

UV‒visible spectral analysis was performed using a Shimadzu UV-1800 PC spectrophotometer (Shimadzu Corporation, Kyoto, Japan). A solution containing crude WSP was diluted 100-fold and subjected to absorption spectrum recording within the wavelength range of 200 to 800 nm [15].

Following the preparation of the WSP extracts, each solution was dried on a rotary evaporator to produce a powder for subsequent analysis. The spectral absorption (FTIR) of the extracts was recorded using a Shimadzu Fourier transform infrared (FT-IR) 8400 s spectrophotometer covering the infrared range of 400–4000 cm^−1^ [15].

Scanning electron microscopy (SEM) was used to study the surface morphology of the WSP extracts. The fixed molecules were washed twice with glutaraldehyde in double distilled water (DDW), dehydrated through a gradient of ethanol from 25% to 100% for 5 min each, and dried overnight in a desiccator. The samples were mounted on the specimen holder using conductive carbon tape and coated with gold using a sputter coater prior to observation using a field emission scanning electron microscope (JEOL JSM-IT 100 model, Tokyo, Japan). Images were taken at different magnifications during this analysis process [16].

### 2.6. Biochemical Polysaccharide Composition

The total and reducing sugar contents of the polysaccharide was calorimetrically measured using the phenol–sulfuric acid and DNS method by drawing a standard curve based on D-glucose according to a previously described method [16,17]. The total sugar content of the polysaccharide was measured spectrophotometrically at an absorbance wavelength of 490 nm. The Bradford method, using the Bio-Rad protein assay, was used to determine the protein content of the polysaccharide, and bovine serum albumin (BSA) was used as the calibration standard [18]. Crude fat content was determined gravimetrically after Soxhlet extraction of the dried samples with hexane [19]. All measurements were carried out in triplicate.

### 2.7. Determination of Monosaccharide Composition

The presence of sugar monomers in EPS was primarily determined as follows: Briefly, 5 mg of WSP stem and leaf polysaccharide samples were hydrolyzed in 4 M trifluoroacetic acid (TFA) in a sealed tube at 100 °C for 2, 4, and 6 h. After hydrolysis, the samples were cooled to room temperature, and the TFA was co-evaporated with methanol to dryness under vacuum. Methanol was added for purification followed by drying, and this cleaning process was repeated 2–3 times. The hydrolyzed WSPRaS and WSPRaL samples and standard sugars (D-glucose (Glc), D-galactose (Gal), D-galacturonic acid (GalA), L-rhamnose (Rha), D-mannose (Man), L-arabinose (Ara), sucrose (Suc), xylose (Xyl), and glucuronic acid (GlcA)) were then analyzed by thin-layer chromatography (TLC) using a silica gel plate (Merck). Sugar solutions were separated on silica gel using a mobile phase in a mixing solution of chloroform, acetic acid, and water (6:7:1). The plate was dried under warm air, sprayed with a mixture of ethanol (95%) and H_2_SO_4_ (5%), and finally heated for 10 min at 105 °C to visualize the colored spots [6]. The retention factor (Rf) represents the ratio of the distance traveled by the saccharide to the distance traveled by the mobile phase during TLC.

### 2.8. High-Performance Liquid Chromatography (HPLC) Conditions

The chromatographic analysis was performed on an Agilent 1100 series instrument (Santa Clara, CA, USA) equipped with various components, including a quaternary pump, a UV‒Vis detector (RID), a manual injector, and LC-Solution software chem32 for data acquisition and processing. The hydrolyzed EPS was separated on a C_18_ column (Aminex HPX-87C HPLC column for sugar analysis 250 mm × 4.0 mm, Bio-Rad, Hercules, CA, USA) with an injection volume of 20 µL. A demineralized water mobile phase was used to separate the sugar monomers at a flow rate of 0.6 mL/min.

### 2.9. Antioxidant and Antibacterial Activities of WSP from the Stems and Leaves of Rhamnus alaternus

#### 2.9.1. In Vitro Antioxidant Activities

The antioxidant activity of water-soluble polysaccharides extracted from the stems and leaves of *R. alaternus* was investigated in detail using several in vitro assays. First, the DPPH radical scavenging activity was assessed according to the method described by Zhou et al. (2023) [16], where the reduction of DPPH radical was measured spectrophotometrically by monitoring the decrease in absorbance at 517 nm after 30 min of incubation in the dark at room temperature. The DPPH radical scavenging capacity was calculated using the following formula: % RSA = [(A_DPPH_ − A_E_)/A_DPPH_] × 100, where RSA is the radical scavenging activity, A_E_ is the absorbance of the antioxidant solution, and A_DPPH_ is the absorbance of the DPPH solution.

The ABTS free radical scavenging activity was then determined using the protocol described by Zhou et al. (2023) [16]. This involved assessing the ability of the polysaccharide extracts to quench the ABTS radical cation, resulting in the percentage inhibition of ABTS^+^ radicals at 734 nm using a Shimadzu UV-type mini-1240 spectrophotometer. The percentage inhibition of ABTS^+^ radicals was calculated using the formula ABTS radical scavenging activity (%) = [(1 − (A/A_0_) × 100], where A_0_ is the absorbance of the control and A is the absorbance of the sample.

In addition, the reducing power of the WSPs was investigated based on their ability to reduce ferric ions according to the method adapted from Zhou et al. (2023) [16]. A blank was prepared with water instead of the sample, and the absorbance of the reaction mixture was measured at 700 nm after 10 min in the dark. This assay measured the formation of the ferric iron ion complex, which is indicative of reducing potential.

Furthermore, the chelating effect on ferrous (Fe^2+^) ions was investigated using the approach proposed by Xu et al. (2021) [20], where the ability of the polysaccharide extracts to bind and sequester ferrous ions was assessed by measuring the decrease in absorbance at 562 nm. The results are expressed as the IC_50,_ and the percentage inhibition of ferrozine-Fe^2+^ complex formation was determined using the following formula: % inhibition = 100 × ((A − (B − C)/A), where A is the absorbance of the control, B is the absorbance without FeCl_2_, and C is the absorbance with FeCl_2_. The control contained FeCl_2_ and ferrozine without extracts.

#### 2.9.2. Antibacterial Activities

Minimum inhibitory concentration (MIC) and minimum bactericidal concentration (MBC)

The MIC and MBC of each WSP extract were determined according to standard protocols [18]. The broth dilution method was used using the same microbial reference strains. Each dilution was incubated with an exponential growth phase bacterial inoculum (A_600_ = 0.4) in the wells of an ELISA plate. The MIC was determined by diluting the extracts in Mueller–Hinton broth, each well containing 10 μL of broth and 10 μL of bacterial inoculum. The initial bacterial concentration was adjusted to 10^6^ CFU/mL. The tested concentrations of the extracts (WSPRaL, WSPRaS) ranged from 0 to 512 μg/mL. Control wells were included for sterility (negative control, no inoculum) and inoculum viability (positive control, no extract). After 24 h of incubation at 37 °C, bacterial growth was assessed by turbidity and pellet formation in the wells. The MIC was defined as the concentration that completely inhibited visible cell growth during the 24 h incubation period. All tests were performed in triplicate.

To determine the MBC values, 10 μL of medium was removed from wells showing no visible growth and plated on Mueller–Hinton agar. After 24 h of incubation at 37 °C, the number of viable organisms was determined as CFU/mL. MBC was defined as the lowest concentration that killed 99% of the bacteria. Each experiment was repeated at least two times [18].

The MIC and MBC determined were characteristic of an extract for a strain tested. Thus, the effect of an extract was considered bactericidal if the MBC/MIC ratio was 1:2. The effect was considered bacteriostatic if the MBC/MIC ratio was =2 [18].

### 2.10. Antibiofilm Activity

The antibiofilm activity of the WSPRaL and WSPRaS extracts was assessed using the serial dilution method on 96-well flat bottom plates (Nunc, Roskilde, Denmark) [21]. Pathogenic strains were cultured overnight in Tryptic soy broth (TSB) (Difco, Franklin Lakes, NJ, USA) broth at 37 °C, then diluted (1:100) in fresh TSB medium with 2% glucose to reach a final OD_600_ of 0.3. In each well, 100 μL of this culture was added, followed by 100 μL of WSPRaL or WSPRaS prepared in TSB containing the tested extracts (6.2–75%). Control wells included TSB medium with glucose and WSPRaL or WSPRaS (negative control) and TSB medium with glucose and bacterial strains (positive control). The plates were then incubated at 37 °C for 24 h to allow biofilm formation. After incubation, the unattached cells were removed by washing each well twice with 200 μL of sterile phosphate-buffered saline (PBS, 7 mM Na_2_HPO_4_, 3 mM NaH_2_PO_4_ and 130 mM NaCl at pH 7.4). The plates were air-dried, followed by staining each well with 150 μL of 1% crystal violet (Merck, Lyon, France) solution in 20% ethanol for 15 min at room temperature. Excess stain was removed by rinsing with sterile water three times. Finally, 200 μL of 30% (*v*/*v*) glacial acetic acid was added to each well, and plates were incubated for 1 h at room temperature. The optical density (OD) at 570 nm (OD_570_) was then measured using a microplate reader (Multiscan FC, Thermo Fisher Scientific, Waltham, MA, USA). All tests were repeated in triplicate, and the biofilm inhibition concentration (BIC) was calculated using the following formula (Equation (2)):

BIC (%) = ((DO_Control_ − DO_Sample_)/DO_Control_) × 100 (2)

### 2.11. Statistical Analyses

The results were analyzed using Statistical Package for the Social Sciences (SPSS) software version 17.0. All values are expressed as the means  ±  standard deviation (SD) from triplicate experiments.

## 3. Results

### 3.1. Extraction and Yield of Water-Soluble Polysaccharides from Rhamnus alaternus

In the last 10 years, the main methods used to extract polysaccharides have included hot water extraction, alkaline solution extraction, microwave-assisted extraction, ultrasonic-assisted extraction, and enzyme-assisted extraction. Hot water extraction has been the traditional method of polysaccharide extraction. Water was used as the solvent for polysaccharide extraction [16].

**Figure 1:**
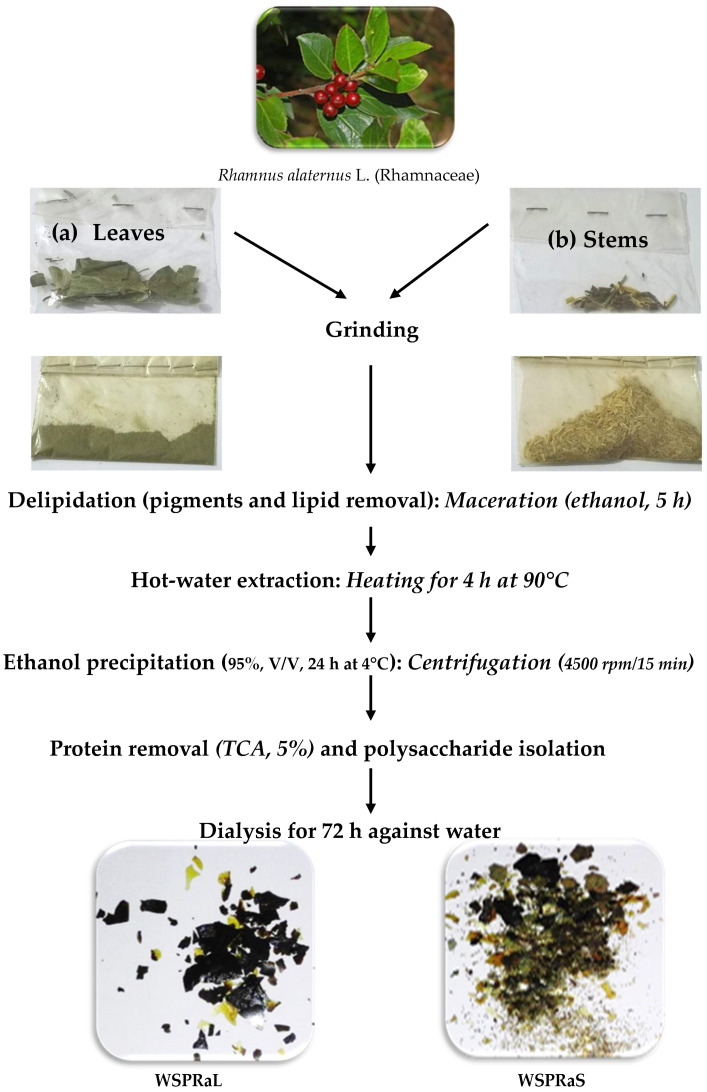
Preparation steps of crude polysaccharides from the leaves (**a**) and stems (**b**) of *Rhamnus alaternus*.

WSP was extracted according to the method described by Bouaziz et al. (2017) [10]. After extraction, the removal of macromolecules and impurities from crude polysaccharides can affect research, production, and application, so further refinement is needed. Polysaccharide removal generally requires proteinization, dialysis, and ethanol precipitation of crude polysaccharides [16]. Using ethanol, the extraction process for WSP from both *R. alaternus* leaves and stems was effective in removing the chlorophyll fraction. This process resulted in a clear powder similar to that of other polysaccharides commonly used in the food industry (Figure 1a,b). After drying at 37 °C, the resulting dry powder exhibited a delightful appearance of ultrafine platelets. The powder obtained after grinding WSPRaS had a brown‒green color (Figure 1b), while that of WSPRaL was olive green (Figure 1a).

The extraction yields of crude polysaccharides from the leaves and stems of *R. alaternus* were approximately 2.774 ± 0.671% and 2.5 ± 0.671%, respectively. Although these extraction rates may appear relatively low, they are consistent with values reported in the literature. In particular, the extraction yields of water-soluble polysaccharides from *Cistanche deserticola* were reported by Ebringerova et al. (1997) [22], Dong et al. (2007) [23], and Wu et al. (2005) [24] to be 25.3%, 1.63%, and 2.1%, respectively. Similarly, the extraction yields from the roots of *Astragalus membranaceus* and the seeds of *Astragalus armatus* were reported to be 0.67% [14] and 4.21%, respectively [25]. In addition, the yield of crude ginger polysaccharide was 2.94% under optimal technological conditions [16]. Furthermore, Li et Zhang (2009) [14] reported an extraction yield of 8.34% for WSP from *Astragalus* sp. roots, while Kiyohara et al. (2010) reported a yield of 1.78% for polysaccharides extracted from the aerial parts of *Astragalus mongholicus* [26]. To increase polysaccharide yield, ultrasound-assisted extraction can be used, as it uses ultrasonic waves to create cavitation bubbles that disrupt plant cell walls, facilitating the release of polysaccharides into the solvent. According to Ebringerova and Hromadkova (2010) [27], optimization of parameters such as ultrasonic power and extraction time can significantly improve extraction efficiency.

### 3.2. Spectral Analysis of Extracted Water Polysaccharides

#### 3.2.1. Ultraviolet Absorption (UV) Spectroscopy

**Figure 2:**
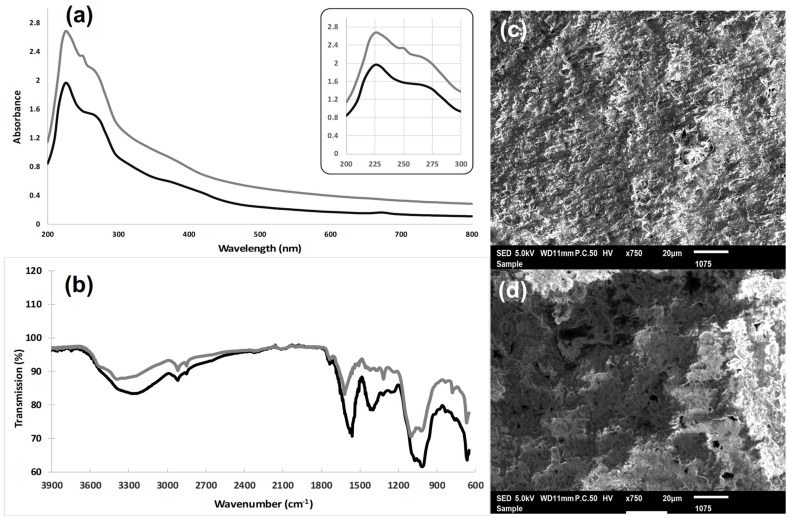
(**a**) UV‒visible and (**b**) FT-IR spectra of stem (**---**) and leaf (**---**) polysaccharides from *Rhamnus alaternus.* SEM images of the surface structures of WSRaPL (**c**) and WSRaPS (**d**) were obtained at a magnification of 5 K (20 μm).

Full-wavelength scanning of the ultraviolet spectrophotometer can be used to determine whether the polysaccharide have been completely removed from the protein and nucleic acid substances, as these impurities have absorption peaks at 260 and 280 nm. Infrared spectroscopy can not only identify the functional groups in polysaccharides but also determine their configuration [16]. The polysaccharide extracts (WSPRaL and WSPRaS) showed a prominent absorbance peak in the ultraviolet region, specifically between 200 and 225 nm. This peak, observed at 205 nm, was attributed to unsaturated carbonyl and carboxyl groups characteristic of water-soluble polysaccharides. Another peak between 245 and 255 nm confirmed the presence of xylose in the polysaccharides tested [28]. Furthermore, the water-soluble polysaccharide extract from *R. alaternus* leaves showed a second peak between 260 and 270 nm, indicating the presence of sucrose and glucose [28]. An additional peak at 675 nm was attributed to the chlorophylls present in the sample (Figure 2a).

#### 3.2.2. Fourier Transform Infrared (FT-IR) Analysis

Fourier transform infrared (FT-IR) spectroscopy has been used to determine the organic functionalities and structural characteristics of carbohydrates [6]. The FT-IR spectra of the water-soluble polysaccharide extracts from the leaves (WSPRaL) and stems (WSPRaS) of *R. alaternus* were analyzed. Absorption bands were assigned to reveal the typical polymeric structure of the carbohydrate (Figure 2b). The spectra showed an intense band between 3500 and 3200 cm^−1^, attributed to the vibration of hydroxyl groups (‒OH) characteristic of polysaccharides, as well as N‒H bonds [16]. This characteristic absorption band of the carbohydrate ring is responsible for the water solubility of WSP. In addition, absorptions corresponding to the C‒H band of CH_2_ and CH_3_ groups and the O‒H band of carboxylic acids were observed at approximately 2914 cm^−1^ and 2847 cm^−1^ for WSPRaS and at approximately 2914 cm^−1^ and 2832 cm^−1^ for WSPRaL. These bands are usually present in hexoses, such as glucose or galactose, or deoxyhexose, such as rhamnose. A prominent peak at 1627 cm^−1^ was observed, which was attributed to the stretching vibration of C=O. This indicated the presence of an aldehyde group (‒CHO) [29]. These results are consistent with previous studies by Venyaminov and Kalnin (1990) [30], where such signals were attributed to primary amide (stretching vibrations of C=O and CN groups) and secondary amide (NH and CN, peptide bonds) bands. In addition, peaks at 1617 cm^−1^ (COO-asymmetric stretch) and 1559 cm^−1^ (COO-symmetric stretch) confirmed the presence of uronic acid [31]. The wavenumber region between 1200 and 800 cm^−1^ is the fingerprint region and can be used to characterize different polysaccharides. Peaks between 1400 and 1200 cm^−1^ are attributed to the C–H variable angle vibration of sugars [16]. The absorbance in the range of 1200 to 1800 cm^−1^ corresponds to proteins and uronic acids, with uronic acids showing characteristic absorbance bands at approximately 1600 and 1420 cm^−1^, indicating asymmetric and symmetric stretching vibrations of the deprotonated carboxylic group (COO^−^). The absorbance peaks at 1418 and 1600 cm^−1^ identified in the IR spectrum of WSPRaL confirm the presence of constitutive uronic acids. The absorption peaks at 1075 and 1070 cm^−1^ in WSPRaL indicated the presence of sulfoxide groups (S=O), confirming the anticoagulant properties of these polysaccharides, as the sulfonyl group reduces blood coagulation [32].

Characteristic absorbance bands of polysaccharides were observed for both WSPRaL and WSPRaS between 1200 and 950 cm^−1^ [33], with the region between 1170 and 980 cm^−1^ corresponding to hydroxyl groups of monosaccharides such as glucose and galactose [34]. Distinct bands for xylose, glucose, and galactose at the pyranose form were identified based on the absorbances of hydroxyl groups and neighboring carbons at C2, C3, and C4 [34]. The peak at 1091 cm^−1^ represented the stretching vibrations of C‒OH bonds, while the band between 1150 and 1160 cm^−1^ indicated the stretching vibrations of C‒O‒C glycosidic bonds involved in the C1 carbon of the cycle [33]. The absorption peak at 844 cm^−1^ indicated that the glucoside linkage in the WSP polysaccharide was α-type [16], as confirmed by the absence of absorbance at 950, 938, and 890 cm^−1^, indicating the presence of β-glycosidic bonds in the polysaccharide [31]. Three strong absorption peaks between 1200 and 1000 cm^−1^ indicated that the sugar ring configuration of the WSP polysaccharides was pyran-type. Infrared spectrum analysis revealed that the WSP polysaccharides had an absorption peak typical of polysaccharides with an α-configuration, and that the sugar ring was a pyran ring [16,31].

#### 3.2.3. Microstructure Analysis

Scanning electron microscopy (SEM) is an observation technique that bridges the capabilities of transmission electron microscopy and optical microscopy. It is widely used to study the surface ultrastructures of various solids, such as polysaccharides, nanomaterials, and metals. To investigate the microstructural characteristics of the WSP polysaccharides, SEM analysis was performed. SEM images (Figure 2c,d) of the water-soluble polysaccharides extracted from the leaves (WSPRaL) and stems (WSPRaS) of *R. alaternus*, observed at 20 μm, revealed that both types of polysaccharides had rough surfaces with well-distributed pores and cavities, resembling a sponge-like structure. This porous morphology is crucial for their water retention capacity. As the number and size of pores increase, so does the water retention capacity, a property highly desirable in various industries such as agriculture, food, cosmetics, and pharmaceuticals [35]. Similar findings were reported by Bouaziz et al. (2017) for polysaccharides and hemicelluloses extracted from almond gum [10]. In addition, Krichen et al. (2016) described porous structures in polysaccharides extracted from the skins of *Ballista capriscus* and *Mustelus mustelus*, which also exhibited water retention properties [36].

### 3.3. Determination of Biochemical Polysaccharide Composition

The biochemical composition of WSP is shown in Table 1. The purified WSP contained total sugars of 482.716 ± 3.02 µg/mg for WSPRaL and 569.135 ± 3.82 µg/mg for WSPRaS, with reducing sugars of 420.240 ± 1.68 µg/mg and 531.732 ± 2.59 µg/mg, respectively. The high total sugar content is consistent with the extraction yield results, indicating that hot water extraction yields the highest total sugar content [37]. Notably, polysaccharides from different parts of the plant showed significant (*p* < 0.05) variations in total sugar content. WSPRaL had a greater total sugar content than WSPRaS, which is consistent with its higher polysaccharide extraction yield, suggesting that leaves, as a medicinal component, contain substantial amounts of polysaccharides. The lower total sugar content in the leaves may be due to the presence of more water-soluble impurities and pigments [37]. Despite the removal of soluble proteins, moderate amounts of proteins were found in both WSPRaL (260.740 ± 0.98 µg/mg) and WSPRaS (269.629 ± 1.48 µg/mg), indicating that both WSPs are acidic polysaccharides and contain small polysaccharide–protein complexes, which often exhibit favorable bioactivities [37]. As shown in Table 1, the lipid content was relatively lower in WSPRaS (13.33 ± 0.28 µg/mg) than in WSPRaL (53.34 ± 2.38 µg/mg).

### 3.4. Determination of the Monosaccharide Composition

#### 3.4.1. Thin-Layer Chromatography (TLC)

Thin-layer chromatography (TLC) is a common and simple method for analyzing polysaccharide composition. This study investigated the hydrolyzed crude polysaccharides from *R. alaternus* stems and leaves by TLC in different hydrolysis time. Indeed, the acid-hydrolyzed sample showed a retention factor similar to the standards used (Table 2). TLC analysis of the crude polysaccharides of the stems revealed the presence of heteropolysaccharides composed of glucose, galactose, xylose, arabinose, and glucuronic acid. Similarly, TLC analysis of the crude polysaccharides of the leaves revealed heteropolysaccharides containing rhamnose, glucose, xylose, arabinose, and galacturonic acid. The polysaccharide composition studied is comparable to that of the crude polysaccharides of pistachio hull, which include rhamnose, glucose, galactose, mannose, xylose, arabinose, and galacturonic acid [6].

The retention factor (Rf) values observed for the sugars analyzed provide insights into their relative mobility during thin-layer chromatography. Based on the Rf values, the sugars can be classified from the most to the least mobile as follows: rhamnose, xylose, arabinose, glucose, galactose, galacturonic acid, and glucuronic acid. The Rf of the monosaccharides was as follow: Rf_Rhamnose_ > Rf_Xylose_ > Rf_Arabinose_ > Rf_Glucose_ > Rf_Galactose_ > Rf_Galacturonic acid_ > Rf_Glucuronic acid_. The TLC results align with the findings presented below. Sucrose was not detected on the TLC plate after 4 and 6 h of hydrolysis, as it had degraded. Its Rf value is 0.19. The migration of sugars in thin-layer chromatography (TLC) is influenced by their polarity, molecular structure, and functional groups. Less polar compounds, like rhamnose, tend to migrate further, as their low polarity reduces interactions with the stationary phase (silica). In contrast, more polar sugars, such as glucose and galactose, are retained more strongly due to their multiple hydroxyl (‒OH) groups, which form hydrogen bonds with the silica. Uronic acids, like galacturonic acid and glucuronic acid, contain a carboxyl (‒COOH) group that increases their polarity and enhances interactions with the stationary phase, significantly limiting their migration. Thus, the polarity and functional groups’ interactions with the stationary phase explain the differences in migration patterns among sugars.

#### 3.4.2. HPLC Separation

**Figure 3:**
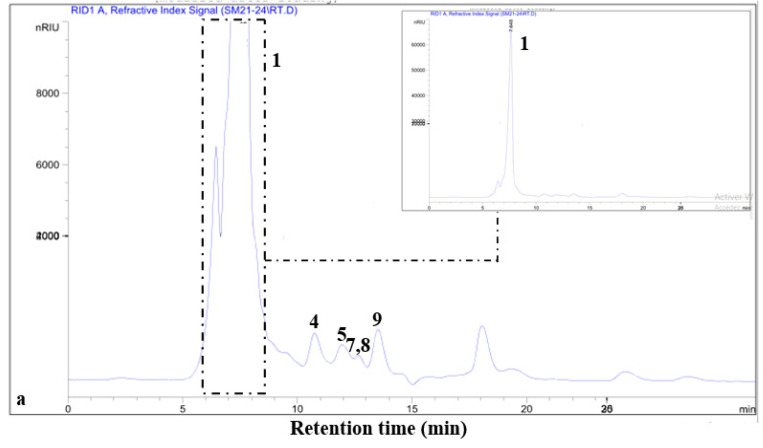

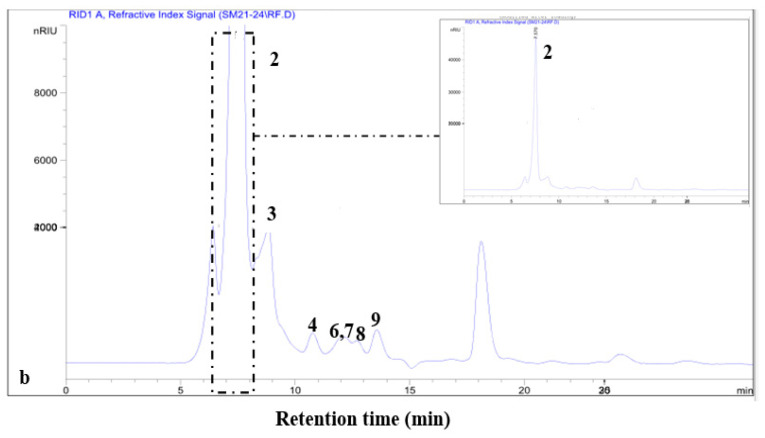
HPLC chromatograms of *Rhamnus alaternus* stem (**a**) and leaf (**b**) polysaccharides recorded at 245 nm. Column: C_18_ Aminex HPX-87C HPLC column for sugar analysis (250 mm × 4.0 mm). Elution (0.6 mL min^−1^) was performed using an isocratic gradient of demineralized water for 40 min, which included (1) glucuronic acid, (2) galacturonic acid, (3) sucrose, (4) glucose, (5) galactose, (6) rhamnose, (7) xylose, (8) mannose, and (9) arabinose.

High-performance liquid chromatography (HPLC) is an effective, quantitative, and comprehensive technique for analyzing carbohydrates [38]. This study optimized HPLC separation conditions for a mixture of nine monosaccharides: mannose, glucose, galactose, rhamnose, xylose, arabinose, glucuronic acid, galacturonic acid, and sucrose. The analysis of polysaccharides purified from *R. alaternus* stems successfully separated the monosaccharides in the following order: glucuronic acid, glucose, galactose, xylose, mannose, and arabinose (Figure 3a). For polysaccharides from *R. alaternus* leaves, the following monosaccharides were separated within 40 min: galacturonic acid, sucrose, glucose, rhamnose, xylose, mannose, and arabinose (Figure 3b). This monosaccharide composition agrees with the results of spectral and TLC analysis.

### 3.5. Antioxidant Activity

#### 3.5.1. DPPH Free Radical Scavenging Activity

A DPPH assay was performed and the reduction of the stable DPPH radical to a yellow molecule was observed after 30 min of incubation with the WSP extracts. The polysaccharides demonstrated scavenging activity that was dependent on the DPPH concentration, showing good antioxidant [6,10].

Polysaccharide extracts from the leaves (WSPRaL) and stems (WSPRaS) of *R. alaternus* exhibited notable DPPH scavenging activity, following a concentration-dependent trend where higher concentrations showed stronger antioxidant effects [6,10]. The elimination capacities of WSPRaS and WSPRaL ranged from 3.45% to 55% and 0.6% to 66%, respectively, at concentrations ranging from 0.5 mg/mL to 7.5 mg/mL. The calculated IC_50_ values for the WSPRaL and WSPRaS extracts were 615 ± 2.05 µg/mL and 628 ± 2.38 µg/mL, respectively, indicating that WSPRaL had the strongest antioxidant activity, while WSPRaS had the weakest (Table 3). The IC_50_ values of WSPRaL and WSPRaS were lower than those reported for other polysaccharides, such as those extracted from almond gum (4 mg/mL), gum hemicellulose (6 mg/mL), guara fruit (10.8 mg/mL), *Prunus cerasus* gum (0.98 ± 0.01 mg/mL) and BHA (0.5 mg/mL) as molecular references [10]. These results suggest that the polysaccharides from the stems and leaves of *R. alaternus* are potent electron donors reacting with free radicals to convert them into more stable products and thereby stabilize radical chain reactions.

#### 3.5.2. ABTS Radical Scavenging Activity

To validate the antioxidant efficiency of the water-soluble polysaccharide extracts from the leaves and stems of *R. alaternus*, which were previously assessed using the DPPH radical scavenging test, we employed a second test based on the proton trapping capacity of the ABTS^°+^ cationic radical. The results demonstrated that the ABTS radical scavenging activity increased with increasing extract concentration. The percentages of polysaccharide extracts inhibited by ABTS are shown in Table 3.

Furthermore, these results indicate that for all concentrations, the polysaccharides from *R. alaternus* leaves exhibited high inhibitory effects than did the stem extract. At a concentration of 1 mg/mL, the water-soluble polysaccharides had inhibitory effects on 79% and 80% of the leaves and stems of *R. alaternus,* respectively. These percentages are significantly higher than the observed value for Trolox, which requires 0.5 mg/mL for 100% inhibition [10]. The IC_50_ values were 470 ± 5.78 µg/mL for WSPRaL and 559 ± 4.32 µg/mL for WSPRaS (Table 3). These IC_50_ values are much lower than those found for polysaccharides from *Lupinus angustifolius* seeds (IC_50_ = 6.98 mg/mL) [39], potato peel polysaccharides (2 mg/mL) [40], and almond gum hemicellulose polysaccharides (5 mg/mL) [10].

In summary, the antioxidant activity determined by the DPPH and ABTS methods does not yield identical results. Therefore, it is imperative to incorporate another test to determine the most potent polysaccharide extract effect.

#### 3.5.3. Reducing Power Assay

Research has revealed a direct correlation between antioxidant activities and reducing power [16]. The determination of reducing power is a crucial technique for understanding the mechanisms of antioxidants action. To measure the reducing power of WSP from the stems and leaves of *R. alaternus*, Fe^3+^-Fe^2+^ transformation was investigated at various concentrations of samples. Although the polysaccharides exhibited low overall reducing power, WSPRaS (IC_50_ = 141.76 ± 3.16 µg/mL) demonstrated a greater reducing effect than WSPRaL (IC_50_ = 203.89 ± 1.07 µg/mL) did at the IC_50_ level. This indicates a close correlation between the reducing power and concentration of WSP, consistent with the findings of Ben Abdallah Kolsi et al. (2017) [41]. The greatest ability to reduce Fe^3+^ to Fe^2+^ ions was observed at a concentration of 100 µg/mL, corresponding to optical density (OD) values of 0.30 and 0.48 for WSRaPS and WSPRaL, respectively. These results are comparable to those obtained for the sulfated polysaccharides of the algae *Porphyra haitanensis*, *Ulva pertusa*, and *Ulva lactuca* and the sulfated polysaccharide from *Cymodocea nodosa*, which showed reducing powers of 0.28, 0.25, 0.33, and 0.3, respectively [41].

#### 3.5.4. Ferrous (Fe^2+^) Ion Chelating Activity

The chelating effects of a compound are significant indicators of potent antioxidant activity. The metal chelating activity test revealed relatively similar results for the water-soluble polysaccharides extracted from the leaves and stems of *R. alaternus* (Table 3). Both extracts exhibited notable Fe^2+^ ion chelating activity, with IC_50_ values of 219 ± 2.51 µg/mL for the leaves and 225 ± 1.75 µg/mL for the stems. However, contrasting results were observed in other studies. For instance, a novel alkali-soluble polysaccharide from *Lepista sordida* (LSAP) demonstrated strong activity with an IC_50_ of 5.14 mg/mL [20]. Additionally, Sila et al. reported metal chelating activity with IC_50_ values of 3.39 mg/mL for pistachio polysaccharides and 0.22 mg/mL for almond polysaccharides. Similarly, Trigui et al. reported that black cumin seeds exhibited an IC_50_ of 0.78 mg/mL [42]. Iron is a well-known pro-oxidant with high reactivity, and Fe^2+^ chelation can involve an antioxidative reaction that retards metal-catalyzed oxidation.

These comparisons underscore the significant chelating capabilities of *R. alaternus* polysaccharides, particularly when they are evaluated against other known sources of polysaccharides. The polysaccharides from *R. alaternus* exhibit competitive Fe^2+^ chelating activity, which is a critical factor in their overall antioxidant potential.

### 3.6. Antibacterial Activities

#### 3.6.1. The Minimum Inhibitory Concentration (MIC) and Bactericidal Concentration (MBC) Determined by the Microdilution Method

The minimum inhibitory concentration (MIC), which is the lowest concentration that stops visible bacterial growth after 18 h of incubation at 37 °C, was calculated using the microdilution method to evaluate the antibacterial activity of the two extracts tested, WSPRaS and WSPRaL. This value characterizes the bacteriostatic effect of the tested extract.

Six reference strains of bacteria were used in this study. The results obtained are summarized in Table 4 and illustrate how the bacterial species and the particular WSP extracts used affected the MIC values. The MICs ranged from 2 to 10 mg/mL, while the MBCs ranged from 4 to 11 mg/mL (Table 4). With a MIC of 2 mg/mL, *Pseudomonas aeruginosa* and *Escherichia coli* showed the highest sensitivity to the WSPRaL extract. In contrast, for *Escherichia coli* and *Klebsiella pneumoniae*, the lowest MIC of the WSPRaS extract was 3 mg/mL. *Bacillus cereus* was the most resistant strain, with MICs for the WSPRaL and WSPRaS extracts of 9 and 11 mg/mL, respectively. The MBC/MIC ratio varied between 1.1 and 2, indicating that the observed growth inhibition was bactericidal.

The strains showed different levels of resistance to the extracts, with the following order for WSPRaL: *Pseudomonas aeruginosa*, *Escherichia coli* > *Enterobacter* sp. > *Staphylococcus aureus* > *Klebsiella pneumoniae* > *Bacillus cereus*. For WSPRaS, the order was *Escherichia coli*, *Klebsiella pneumoniae* > *Pseudomonas aeruginosa*, *Enterobacter* sp. > *Staphylococcus aureus* > *Bacillus cereus*. In particular, *Bacillus cereus* showed the highest resistance, with MICs of 10 mg/mL and 9 mg/mL for WSPRaS and WSPRaL, respectively.

In addition to MIC testing, the antibacterial activity was further confirmed by measuring inhibition zones. The WSPRaL extract showed significant activity against both *P. aeruginosa* and *Bacillus cereus*, with an inhibition zone diameter of 15 mm. The WSPRaS extract showed particularly strong antibacterial activity against *P. aeruginosa*, with a zone of inhibition (ZOI) diameter of approximately 16 mm. However, *Bacillus cereus* remained the most resistant strain, with a ZOI diameter of only 5 mm. These results highlight the potent antibacterial activity of the WSPRaS extract against *P. aeruginosa*.

In conclusion, the results suggest that the extract of water-soluble polysaccharides from the leaves of *R. alaternus* (WSPRaL) showed the most potent activity against the reference strains tested. Overall, both polysaccharides show significant potential to inhibit bacterial growth and could be considered alternatives to conventional antibiotics and pharmaceuticals. Polysaccharides exhibit specific antibacterial activity against both Gram-negative and Gram-positive bacteria [43]. They are readily accessible, nontoxic, and have great potential for widespread use as novel antibacterial agents in medicine and food. The mechanisms underlying the antibacterial activity of polysaccharides are complex. Polysaccharides exert their antibacterial effects primarily by compromising cell membrane and cell wall integrity, inhibiting biofilm formation, affecting bacterial metabolism, disrupting protein synthesis, and inhibiting nutrient uptake [43].

#### 3.6.2. Biofilm Inhibition Activity

Biofilm formation is a major global health challenge, leading to a focus on preventive strategies. The use of medicinal plants is emerging as an alternative approach for the treatment of various infectious diseases. Naturally occurring polysaccharides have been recognized for their antimicrobial potential activities against various bacterial pathogens by disrupting biofilm formation, quorum sensing, efflux pumps, bacterial cell wall and membrane synthesis, and bacterial nucleic acid synthesis [44].

**Figure 4:**
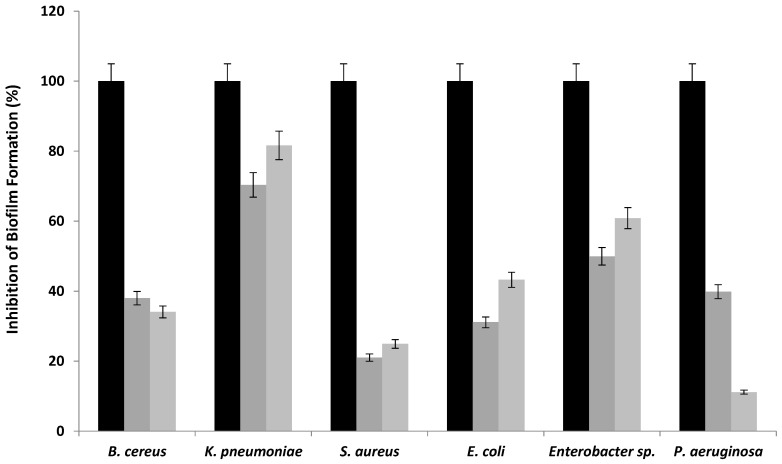
Effect of WSPRaL (■) and WSPRaS (■) on the binding of pathogenic bacteria expressed as a percentage of inhibition evaluated by the crystal violet test (positive control (■)).

The antibiofilm activity of WSP extracted from the leaves and stems of *R. alaternus* was evaluated against pathogenic bacteria. Remarkably, these polysaccharide extracts exhibited significant inhibition of biofilm formation against all bacteria tested (Figure 4), with inhibition percentages ranging from 21 to 70% for WSPRaL and from 11 to 81% for WSPRaS. In particular, WSP from *R. alaternus* leaves inhibited biofilm formation by 70% against *K. pneumoniae* at a concentration of 5 mg/mL. However, the inhibition percentages for the other strains did not exceed 50%. WSP from *R. alaternus* stems inhibited biofilm formation by 81% and 62% against *K. pneumoniae* and *Enterobacter* sp. at concentrations of 3 and 4 mg/mL, respectively. Notably, *P. aeruginosa* was less sensitive, with biofilm inhibition percentages not exceeding 11%.

The variation in inhibition percentages among the polysaccharides tested suggested differences in the composition and potency of each extract. Their antibiofilm formation is likely to be mediated by mechanisms beyond growth inhibition, including biosurfactant properties that alter bacterial–surface interactions, modulation of gene expression in bacteria, and competitive inhibition of carbohydrate–protein interactions. Thus, antibiofilm polysaccharides formation could block lectins or sugar-binding proteins present on the surface of bacteria or block tip adhesins of fimbriae and pili. For example, the lectin-dependent adhesion of pathogenic *P. aeruginosa* to human cells is efficiently inhibited by galactomannans [45]. Plant polysaccharides are therefore highly regarded for their safety and nontoxicity. Most plant polysaccharides exhibit various biological properties, including antibacterial, antioxidant, immunomodulatory, and health benefits [43]. Plant polysaccharides from various sources are typically extracted using hot water. The inherent activity of plant polysaccharides is usually low. Most plant polysaccharides have low intrinsic activity [43].

## 4. Conclusions

In this study, polysaccharides were successfully isolated from the leaves (WSPRaL) and stems (WSPRaS) of *Rhamnus alaternus* with yields of 3% and 3.25%, respectively. Characterization revealed that WSPRaL and WSPRaS was composed of proteins, lipids, total sugars, and reducing sugars. FTIR, TLC, and HPLC analyses identified the monosaccharide components, with WSPRaS consisting mainly of glucuronic acid, glucose, galactose, xylose, mannose, and arabinose, and WSPRaL comprising galacturonic acid, sucrose, glucose, rhamnose, xylose, mannose, and arabinose. SEM was used to determine the microstructure of the polysaccharides, and FTIR and UV–visible spectroscopy further characterized their physicochemical properties. Antioxidant activities of WSPRaL and WSPRaS were evaluated using DPPH, ABTS radical scavenging, reducing power, and chelating capacity assays, with both polysaccharides displaying significant activity. Additionally, the polysaccharides showed notable antibacterial and antibiofilm effects against pathogenic strains. Collectively, these results demonstrate the potential of *R. alaternus* polysaccharides as bioactive natural agents, offering valuable antioxidant and antimicrobial properties. Their promising applications span various sectors, including the food, cosmetic, pharmaceutical, and medical industries, underscoring their versatility as functional and bioactive compounds.

## Figures and Tables

**Table 1:** Chemical composition and monosaccharide composition of WSPRaL and WSPRaS.

	WSPRaL	WSPRaS
	Chemical Composition	
Proteins (µg/mg)	260.740 ± 0.98	269.629 ± 1.48
Lipids (µg/mg)	53.34 ± 2.38	13.33 ± 0.28
Total sugar (µg/mg)	482.716 ± 3.02	569.135 ± 3.82
Reducing sugar (µg/mg)	420.240 ± 1.68	531.732 ± 2.59
	Saccharide composition (%)	
Glucuronic acid	-	89.865 ± 2.42
Galacturonic acid	77.373 ± 2.34	-
Sucrose	13.131 ± 1.9	-
Glucose	2.336 ± 0.45	2.765 ± 0.66
Galactose	-	2.162 ± 0.52
Rhamnose	2.936 ± 0.24	-
Mannose	0.742 ± 0.08	0.503 ± 0.04
Xylose	0.740 ± 0.06	0.5 ± 0.02
Arabinose	2.740 ± 0.48	2.662 ± 0.76

**Table 2:** Retention factor of the standard monosaccharides used to determine the composition of WSPRaL and WSPRaS using TLC.

Monosaccharide	Rf	
	WSPRaL	WSPRaS
Rhamnose	0.56	-
Xylose	0.45	0.45
Arabinose	0.34	0.34
Glucose	0.27	0.27
Galactose	-	0.23
Galacturonic acid	0.14	-
Glucuronic acid	-	0.11

**Table 3:** Total antioxidant activities of WSPRaL and WSPRaS polysaccharides.

	IC_50_ (DPPH)	IC_50_ (ABTS)	IC_50_ (FRAP)	IC_50_ (Chelating Power)
RaWSPL	615 ± 2.05 µg/mL	470 ± 5.78 µg/mL	203.89 ± 1.07 µg/mL	219 ± 2.51 µg/mL
RaWSPS	628 ± 2.38 µg/mL	559 ± 4.32 µg/mL	141.76 ± 3.16 µg/mL	225 ± 1.75 µg/mL

**Table 4:** CMI of the WSPRaL and WSPRaS extracts.

Strains	MIC (mg/mL)		MBC (mg/mL)		MBC/MIC	
	WSPRaL	WSPRaS	WSPRaL	WSPRaS	WSPRaL	WSPRaS
*Bacillus cereus*	9	10	11	11	1.22	1.1
*Klebsiella pneumoniae*	5	3	7	5	1.4	1.66
*Staphylococcus aureus*	4	5	6	7	1.5	1.4
*Escherichia coli*	2	3	4	6	2	2
*Enterobacter* sp.	3	4	5	7	1.66	1.75
*Pseudomonas aeruginosa*	2	4	4	6	2	1.5

## Data Availability

All data and materials are available upon request.
